# Photo-Induced Force Microscopy by Using Quartz Tuning-Fork Sensor

**DOI:** 10.3390/s19071530

**Published:** 2019-03-29

**Authors:** Junghoon Jahng, Hyuksang Kwon, Eun Seong Lee

**Affiliations:** Center for Nanocharacterization, Korea Research Institute of Standards and Science, Daejeon 34113, Korea; phyjjh@kriss.re.kr (J.J.); h.kwon@kriss.re.kr (H.K.)

**Keywords:** photo-induced force microscopy (PiFM), quartz tuning fork, nano-optics

## Abstract

We present the photo-induced force microscopy (PiFM) studies of various nano-materials by implementing a quartz tuning fork (QTF), a self-sensing sensor that does not require complex optics to detect the motion of a force probe and thus helps to compactly configure the nanoscale optical mapping tool. The bimodal atomic force microscopy technique combined with a sideband coupling scheme is exploited for the high-sensitivity imaging of the QTF-PiFM. We measured the photo-induced force images of nano-clusters of Silicon 2,3-naphthalocyanine bis dye and thin graphene film and found that the QTF-PiFM is capable of high-spatial-resolution nano-optical imaging with a good signal-to-noise ratio. Applying the QTF-PiFM to various experimental conditions will open new opportunities for the spectroscopic visualization and substructure characterization of a vast variety of nano-materials from semiconducting devices to polymer thin films to sensitive measurements of single molecules.

## 1. Introduction

Combining scan probe techniques with optical illumination is a powerful approach in nano-imaging technology to add spectroscopic sensitivity to the nanoscopic resolution provided by a sharp tip. A new and emerging technique in this area is photo-induced force microscopy (PiFM) [1,2], which enables the spectroscopic probing of materials with a spatial resolution well under 10 nm. The contrast in PiFM is directly related to the optical properties of the sample underneath the tip and can, in principle, be conducted in noncontact/tapping mode atomic force microscopy (AFM). One of the advantages of this approach is a far-field noise-free detection so that it shows a superior sensitivity to the light detection-based nano-optic measurement techniques such as scanning nearfield optical microscopy (SNOM). The PiFM approach can be used for probing optical fields near surfaces [3] and nanostructures [4,5], as well as for detecting spectroscopic transitions in the sample [6,7,8]. The photo-induced forces measured in PiFM act in a spatially confined region on a nanometer scale, which translates into a very high spatial resolution even under ambient conditions. Moreover, the PiFM approach is compatible with a wide range of optical excitation frequencies from the visible [9] to the mid-infrared [10,11], enabling the nanoscale imaging of various contrast mechanisms based on either electronic or vibrational transitions in the sample.

Despite this promising feature making PiFM an attractive method for the characterization of nanomaterials, it is still a challenge to install the system in various environmental conditions such as the vacuum chamber or the liquid system because of the complex beam bouncing optics to monitor the probe motion. Replacing the conventional cantilever probe with a quartz tuning fork (QTF) can be a solution because the QTF is a self-sensing sensor, which does not require the complex beam bouncing optics [12]. In this article, we demonstrate the nano-optical imaging performance of the quartz tuning fork-based photo-induced force microscopy by compactly configuring the system with the versatile light coupling modes for the transparent (bottom illumination) and the opaque (side illumination) samples.

## 2. Materials and Methods

### 2.1. Photo-Induced Forces

**Figure 1:**
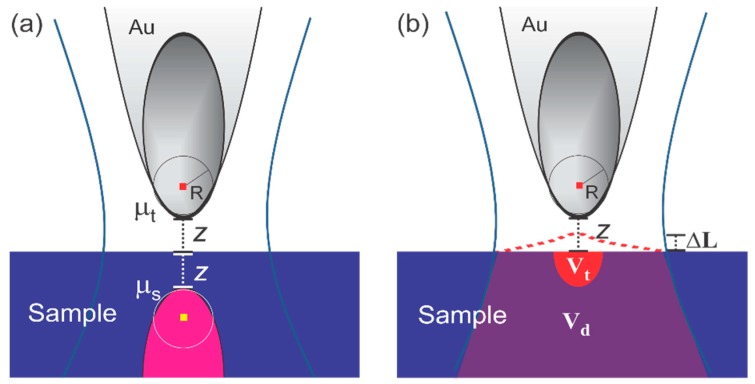
Diagrams of (**a**) the field gradient and scattering force and of (**b**) the thermal expansion force: μ_t_ and μ_s_ are the induced dipoles on the tips and samples, respectively. V_t_ is the absorption volume due to the tip-enhanced field, and V_d_ is the absorption volume due to the transmitted light.

When the light illuminates the tip-sample junction, two kinds of photo-induced forces are manifested. One is the optical field gradient and scattering force [2], and the other one is the thermal expansion force mediated by the intermolecular interaction between the tip and the sample [7]. The schematic diagrams of the two kinds of force interactions are sketched in Figure 1a,b respectively. 

#### 2.1.1. Optical Field Gradient and Scattering Force

The incident light induces a dipole on the tip, which then mutually interacts with the reflected field from the sample. The mechanisms are illustrated in a simplified picture in Figure 1a. When the field near the tip is *E*, an optical field gradient and scattering force on the tip is given as Refs. [2,13]

(1) $$F_{\mathrm{pif}}\approx -\frac{\alpha_{t}^{\prime }}{2}\nabla {\left|E\right|}^{2}+\omega \alpha_{t}^{\prime \prime }\langle E\times B\rangle ,$$

where $\alpha_{t}$ and $\alpha_{s}$ are the complex effective polarizabilities of the tip and sample, respectively, within the point dipole approximation; $\alpha_{t}^{\prime }$ and $\alpha_{t}^{\prime \prime }$ are the real and imaginary part of the tip’s polarizability; and *z* is the distance from the tip end to the surface. The field *E* consists of the incident field and the nearfield at the tip–sample junction. In a tightly focused beam case, the long-range gradient force from the first term in Equation (1) is generated due to the strong z-dependence of the incident field. However, it becomes ignorable at the center of the focal spot where the incident field shows approximately a uniform field distribution. Then the total force at the tip end becomes

(2) Fpif≈−3Re{αt*αs}2πε0(2z)4|E0|2+ωαt″〈E0×B0〉.

where the *E*_0_ is the incident field. The first term in Equation (2) is the short-range attractive force due to the multi-reflection between the tip and the sample, which can be analyzed by the image dipole method with the sample information. The second term is the long-range repulsive scattering force, which is based on the direct light–tip interaction. This force formula is also applicable to the side-illumination geometry by replacing the *E*_0_ with *E*_0_ cosθ, where the θ is the incident angle. The short-range force strongly increases with the *z*^−4^ dependence as the tip approaches the sample. In classical nearfield scattering theory, $\alpha_{s}$ can be successfully explained with the help of an image dipole model, which yields *α_s_* = *β α_t_*, where *β* is the complex electrostatic reflection coefficient, given as *β* = ε − 1 / ε + 1, where ε is the dielectric constant of the sample. This short-range-induced dipole force typically shows the dispersive spectral line shape and strongly increases in the metal/plasmonic material where ε is negative, whereas the force is typically small under a few pN range in the organic and inorganic sample where ε is positive, even at its molecular resonance [14,15,16,17].

#### 2.1.2. Thermal Expansion Force Mediated by Intermolecular Interaction between Tip and Sample

Near molecular resonances, there is a strong light absorption of the sample followed by a temperature rise, which results in the strain deformation of the sample that eventually gives rise to the thermal expansion. The thermal expansion generates a force, which is the result of several causal processes: First, there is an energy exchange between the sample and the light field, which scales with the optical absorption coefficient and results in a temperature rise (ΔT ~ P_abs_). The strong field-enhancement near a sharp metal tip can further increase the absorption of the sample under the tip. Second, the accumulated heat diffuses to deform the sample to induce a thermal expansion (ΔL ~ ΔT). Third, the thermal expansion changes the tip–sample distance, which introduces a modulation of the intermolecular force between the tip and the sample (ΔF ~ ΔL). The force is given in Ref. [7]:

(3) $$F_{\mathrm{pif}}\approx \frac{\partial F_{\mathrm{ts}}}{\partial z}{\Delta L}_{\mathrm{tot}}\left(z\right),$$

where ΔL_tot_ is the total thermal expansion of the sample and *F*_ts_ is the intermolecular force which is typically made up of the attractive force in the noncontact region and the repulsive force in the contact region [18]. Because the force is based on the absorption, the force spectrum follows the dissipative line shape. There are two kinds of thermal expansion of the sample underneath a sharp metal tip. These mechanisms rely on the fact that the sample experiences two kinds of optical fields; one is the incident laser field, and the other one is the tip-enhanced nearfield. The direct thermal expansion (ΔL_d_), which absorbs the incident light, is irrelevant to the presence of the tip and, thus, exhibits a continuous increase with the sample thickness. On the other hand, because the field-enhancement at the tip–sample junction is limited by the extent of the nearfield underneath the tip and decreases with increasing sample thickness on high-index substrates such as Si, the tip-enhanced thermal expansion (ΔL_t_) increases to a certain sample thickness and eventually decreases, i.e., it shows a maximum [7]. The expansion mechanisms are illustrated in a simplified picture in Figure 1b. This force is typically in the range between a few tens of pN to a few hundreds of pN. Thus, on the molecular resonance, the thermal expansion force overwhelms the short-range-induced dipole force.

#### 2.1.3. PiFM Sideband Coupling Dynamics

The PiFM sideband coupling dynamics can be understood as a bimodal AFM operation, where the probe is driven by two external driving sources, a piezo ditherer (*f*_2_) and a light modulation (*f*_m_ = *f*_2_ − *f*_1_). The probe motion is expressed as

(4) $$\underset{i=1,2}{\Sigma }m{\overset{..}{z}}_{i}+b_{i}{\dot{z}}_{i}+k_{i}z_{i}\approx F\left(t\right),$$

with $F\left(t\right)=F_{2}\cos\left(2\pi f_{2}t\right)+F_{\mathrm{pif}}\left(z\right)\cos\left(2\pi f_{m}t\right)+F_{\mathrm{ts}}\left(z\right)$ and $z_{i}=A_{i}\sin\left(2\pi f_{i}t+\theta_{i}\right)$, where *i* is the *i*th eigenmode of the probe, *z_i_* is the *i*th sinusoidal motion with amplitude *A_i_* and phase *θ_i_*, *z* ≈ *z*_1_ *+ z*_2_, *F*_2_ is the force from the external piezoelectric actuator to mechanically dither the quartz tuning fork, and *F*_ts_ is the intermolecular force between the tip and the sample. The PiFM sideband-coupled force acting on the probe at the fundamental eigenmode (*f*_1_) is given in Ref. [19]:

(5) $$F(f_{1})\sim \frac{\partial F_{pif}(f_{2}-f_{1})}{\partial z}A_{2}(f_{2}).$$

The tapping amplitude, which is demodulated at the second mechanical resonance (*i* = 2), is sideband-coupled with the photo-induced force gradient and carried it to the fundamental mechanical resonance (*i* = 1). The PiFM amplitude with a sideband-coupled mode, which is demodulated at *f*_1_, depends on the derivative of the photo-induced force rather than the force F_pif_ itself. Thus, the constant forces such as the scattering force in Equation (1) and the thermal expansion force based on direct thermal expansion in Equation (2) are excluded with the PiFM sideband mode.

### 2.2. PiFM Measurements

#### 2.2.1. Instruments

**Figure 2:**
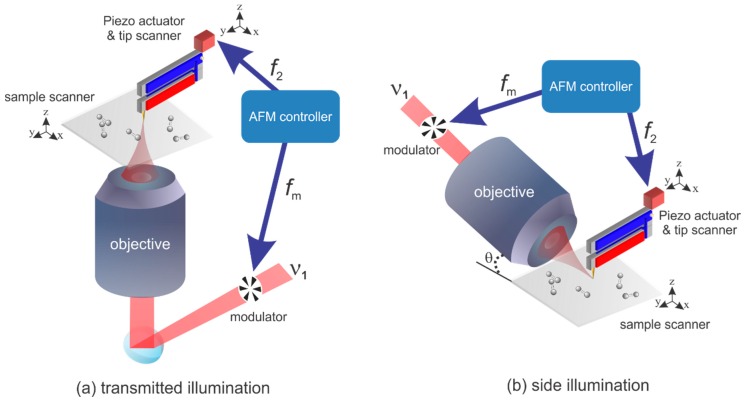
Sketches of the quartz tuning fork-based photo-induced force microscopy with (**a**) transmitted and (**b**) side illumination.

The MultiView 2000 and MultiView 4000 tuning fork-based AFM system from Nanonics Imaging Ltd. (Jerusalem, Israel) are customized for the PiFM experiment. The MultiView 2000 is coupled to an 800-nm CW laser for the transmission system. The laser beam illuminated the sample in an inverted microscope equipped with a high-numerical-aperture (NA = 1.25) objective lens as sketched in Figure 2a. The MultiView 4000 is coupled to a 660-nm CW laser with the side illumination geometry. The laser beam illuminated the sample around 40 degrees with a long working distance objective (NA = 0.6) as sketched in Figure 2b. In the rest of the experiments, the average illumination power for the two lasers are around 400–800 μW. The microscope is operated in tapping mode with a commercial gold-coated tuning fork from Nanonics Imaging Ltd. Typically the fundamental resonance is around 32 kHz, and the second mechanical resonance is around 190 kHz. To gain a higher PiFM signal to noise ratio, we use the second resonance for the topography feedback and the fundamental resonance for the PiFM demodulation with an external lock-in (7280 DSP lock-in, Signal Recovery). The laser intensity is modulated by using an accusto-optical modulator at the frequency of f_m_ which sets to the difference frequency between the fundamental and the second resonance (*f*_m_ = *f*_2_ − *f*_1_ = 158 kHz) for the sideband operation. For the PiFM demodulation in the lock-in amplifier at the fundamental resonance, the frequency mixer couples the driving frequency (*f*_2_) with the light modulation frequency (*f*_m_) to generate the reference signal (*f*_1_). Both MultiView AFM systems have two kinds of scanners: one is the tip scanner in which the sample stage is fixed and the tip scans over the sample. The other one is the sample scanner in which the tip is fixed and then the sample moves. In the PiFM operation, the tip scan finds the focal spot and parks the tip at the center of it. Then, the sample scan is used for the PiFM imaging of the sample.

#### 2.2.2. Sensitivity of a Probe in PiFM 

The minimum detectable force of a QTF is derived from the thermal noise of the *i*th eigenmode of the QTF which is given as $N_{i}=\sqrt{4K_{B}TBQ_{i}/\omega_{i}k_{i}}$, where *k*_i_, *Q*_i_, and *ω*_i_ are the stiffness, quality factor, and angular resonance frequency of the *i*th eigenmode of the QTF; *K*_B_ is the Boltzmann constant; *B* is the system bandwidth; and *T* is the absolute temperature. For our tuning-fork parameters given as *ω*_1_ = 2 π × 32.3 KHz, *Q*_1_ = 1125, *k*_1_ = 2000 N/m, *ω*_2_ = 2 π × 188.5 KHz, *Q*_2_ = 613, and *k*_2_ = 7.8 × 10^5^ N/m, the thermal noises are $N_{1}\approx 0.21\;\mathrm{pm}$ and $N_{2}\approx 0.01\;\mathrm{pm}$ for the fundamental and the second eigenmodes respectively, where T = 300 K and B = 1 Hz. The minimum detectable forces, $F_{\min_{i}}=k_{i}N_{i}$, are 0.38 pN and 1.33 pN for the fundamental and the second eigenmodes, respectively. Because the second eigenmode is less sensitive than the fundamental eigenmode, we use the fundamental resonance for the PiFM demodulation and the second resonance for the feedback. 

### 2.3. Sample Preparation

The diluted Silicon 2,3-naphthalocyanine bis(trihexylsilyloxide) (SiNc) in the toluene from Sigma Aldrich Inc. (St. Louis, MO, USA) is spin-coated onto the glass substrate. It resulted in various sizes of molecular clusters from a few nm to a few hundred nm. The graphene layered sample is prepared by mechanically exfoliating a graphite and transferring it to a Si substrate with the 3M tape method which resulted in various layers of graphene from a monolayer to a few tens of layers.

## 3. Results

**Figure 3:**
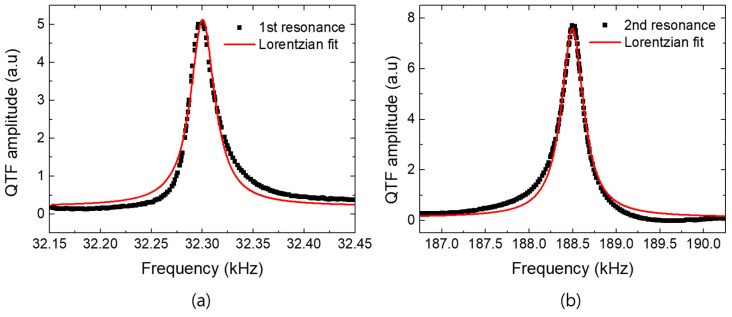
The frequency sweeping of the quartz tuning-fork sensor near (**a**) the fundamental resonance and (**b**) the second resonance by using the external piezo actuator.

By using the mechanical piezo actuator, the fundamental and the second resonance curves of the quartz tuning fork are measured in Figure 3a,b respectively. As reported in Ref. [20], the mechanical piezo actuator is better to excite the higher eigenmodes of the QTF than the electrical self-oscillation. The measured results (black dots) are well-fitted by the Lorentzian curve (red solid line), which presents the resonance and quality factor of 32.3 kHz and 1125 for the fundamental eigenmode and of 188.5 kHz and 613 for the second eigenmode respectively. Because the quality factor of the second eigenmode is smaller than the fundamental, to gain a higher PiFM signal-to-noise ratio, the second resonance is used for the topography probing and the fundamental resonance demodulates the PiFM amplitude with the sideband mode. 

**Figure 4:**
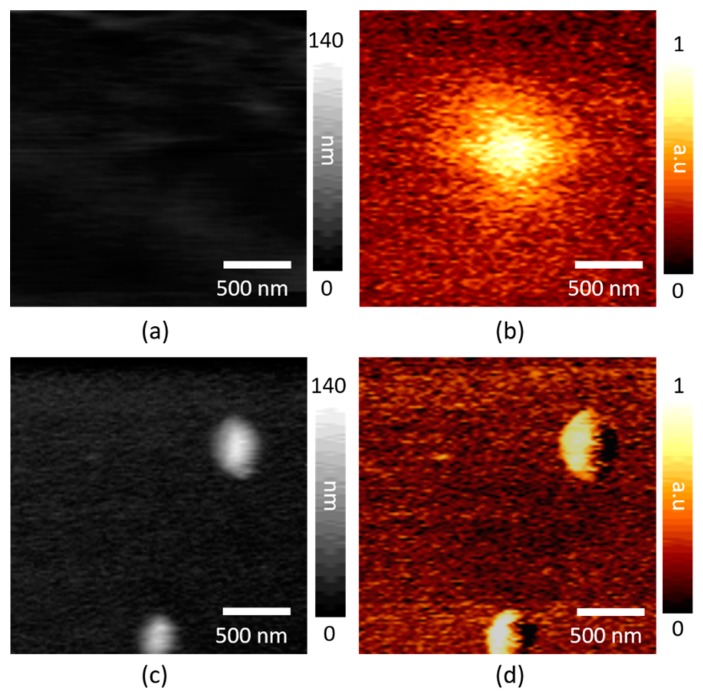
The photo-induced force microscopy (PiFM) mapping of the SiNc molecular clusters on a glass substrate at 800 nm: (**a**) The topography and (**b**) PiFM image of SiNc by the tip scanner to find the focal spot and (**c**) the topography and (**d**) PiFM image of SiNc by the sample scanner after parking the tip at the center of the spot in **b**.

The SiNc molecular clusters are mapped in Figure 4. The tip scanner visualizes the focal spot by crossing over the focused beam on the clean glass substrate area in Figure 4b with the simultaneously measured topography in Figure 4a. The full-width half maximum (FWHM) of the focal spot is around 350 nm. After parking the tip at the center of the spot, the SiNc clusters are successfully visualized by using the sample scanner in Figure 4d. Compared to the simultaneously measured topography in Figure 4c, the PiFM shows a better signal-to-noise ratio because it follows the derivative of the photo-induced force.

**Figure 5:**
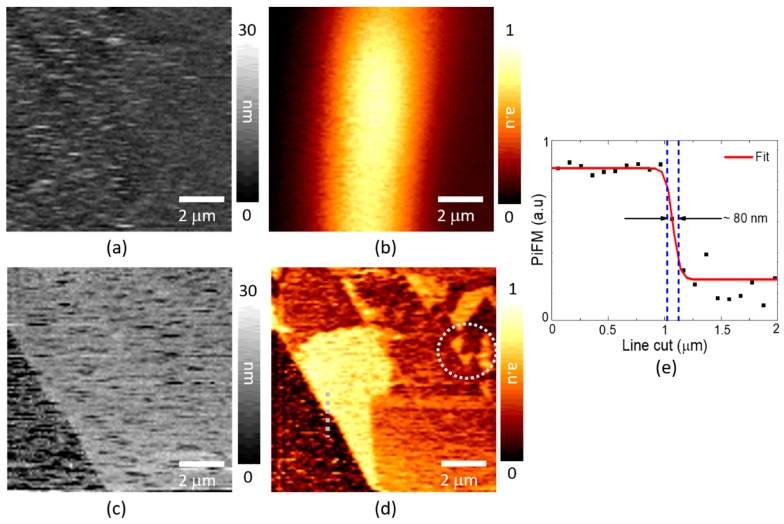
The PiFM mapping of graphene on an Si substrate at 660 nm: (**a**) The topography and (**b**) PiFM image of graphene by the tip scanner to find the elongated focal spot and (**c**) the topography and (**d**) PiFM image of graphene by the sample scanner after parking the tip at the center of the spot in Figure 5b. (**e**) The estimated spatial resolution of our QTF-based PiFM.

For the opaque sample, the side-illumination geometry is applicable rather than the transmission geometry. When the light illuminates the sample from the side, the beam shape is elongated at the surface. The tip scan image on the clean Si substrate visualizes the elongated focal spot well in Figure 5b. Then, the tip parks at the center of the spot, and now, the sample scan visualizes the graphene layers in Figure 5c,d. The spatial resolution of our QTF-based PiFM measurement is estimated under 80 nm from the gray dashed line cut of the Graphene in Figure 5d, which is replotted in Figure 5e. An interesting observation is found in Figure 5d, where the PiFM image shows totally different contrasts to the topography measurement in Figure 5c. Especially the circled region in Figure 5d is composed of several irregularly patterned patches while it is seemingly uniform in thickness in topography. These dramatically different images might possibly be explained by the self-assembled environmental adsorbates of thickness 0.3 nm on the graphene surface [21]. According to Ref. [21], the bright and dark regions have different orientations of chain-like organic molecules of which the polarization responses are different.

## 4. Discussion

The focal spots in Figure 4b and Figure 5b are measured by using the far-field gradient force in Equation (1), which is mostly the direct light–tip interaction that depends on the shape of the focal volume. In the tightly focused beam case in Figure 4b, the focal spot of the 800-nm wavelength beam has the Gaussian distribution which measured around of a 350 nm diameter (FWHM) by using the tip scanning. This corresponds well to the theoretical diffraction limit, given as λ/2NA ≈ 320 nm where NA is 1.25. In the side illumination geometry, the shape of the focal spot shows the elongated ellipsoid in the micrometer scale. After parking the tip at the center of the spots, the short-range tip–sample interaction chemically characterizes the samples. Because the short-range-induced dipole force is close to our noise level (approximately under a few pN) in the positive permittivity, the tip-enhanced thermal expansion force dominates in the PiFM chemical characterization of the SiNc at the 800-nm absorption as well as the graphene at the 660-nm absorption. The PiFM sideband mode, which is the force gradient measurement, shows a better contrast than the topography because it extracts the localized force by reducing the constant background forces.

## 5. Conclusions

In sum, we present the quartz tuning fork as a force sensor in the photo-induced force microscopy. The self-sensing ability with the high stiffness of the QTF helps to compactly configure the PiFM system without the jump-to-contact issue. We demonstrate that the system can be utilized with respect to the angle of the coupled light, which applies to the transparent and opaque sample respectively. Applying PiFM to various environmental systems will open new opportunities for the spectroscopic visualization and substructure characterization of a vast variety of nano-materials with a functionalized tip [22], from semiconducting nanoparticles to polymer thin films to sensitive measurements of single molecules.
